# Effects of fish oil on serum lipid profile in dialysis patients: a systematic review and meta-analysis of randomized controlled trials

**DOI:** 10.1186/1476-511X-13-127

**Published:** 2014-08-08

**Authors:** Wei Zhu, Chongya Dong, Han Du, He Zhang, Jie Chen, Xiaohong Hu, Feng Hu

**Affiliations:** Department of Nephrology, The 150th Hospital of PLA, Luoyang, China; Department of Epidemiology and Biostatistics, School of Public Health, Peking University, Beijing, China; Department of Nephrology, Kidney Institude of PLA, Changzheng Hospital, Second Military Medical University, Shanghai, China

**Keywords:** Fish oil, Dialysis, Lipid, Meta-analysis

## Abstract

**Background:**

The effects of fish oil supplements on lipid profile in dialysis patients are controversial. With increasing interest in the potential health benefits of fish oil, it is important to explore its real effects.

**Objective:**

We aimed to identify and quantify the effects of fish oil on triglyceride (TG), total cholesterol (TC), high-density lipoprotein cholesterol (HDL-C), and low-density lipoprotein cholesterol (LDL-C) in dialysis patients.

**Methods:**

PubMed, EMBASE and the Cochrane Central Register of Controlled Trials were searched for relevant trials of fish oil and lipid profile in dialysis patients. We identified 209 potential studies and included 13 randomized controlled trials. Eligible studies, determined by consensus using predefined criteria, were reviewed in accordance with the Preferred Reporting Items for Systematic Reviews and Meta-Analyses (PRISMA) guidelines and a meta-analysis was performed.

**Results:**

Compared with the control group, serum TG and TC levels in the fish oil group were reduced by 0.23 mmol/L (95% CI, −0.31, −0.14, *P* <0.01) and 0.12 mmol/L (95% CI, −0.23, −0.01, *P* =0.03), respectively. HDL-C levels were increased by 0.20 mmol/L (95% CI, 0.01, 0.40, *P* <0.01) attributable to fish oil. In contrast, fish oil did not influence serum LDL-C levels. Subgroup analysis showed the effects of fish oil were stronger in subjects with higher baseline TG levels, and the long-term intervention (>12w) demonstrated a tendency towards greater improvement of serum HDL-C and LDL-C levels compared with short-term intervention (≤12 w). However, both of the changes were not statistically significant in meta-regression analysis. There were no obvious difference in effects of different doses and components of fish oil on lipid levels.

**Conclusion:**

Fish oil supplements reduced serum TG and TC levels, and increased HDL-C levels, without affecting LDL-C levels among dialysis patients. It should benefit patients at risk of cardiovascular diseases. Based on randomized controlled trials, we suggested a daily supplement dose of fish oil for dialysis patients of >1 g, but a high dose might not be necessary.

**Electronic supplementary material:**

The online version of this article (doi:10.1186/1476-511X-13-127) contains supplementary material, which is available to authorized users.

## Introduction

Dialysis patients have an inordinate risk of cardiovascular disease (CVD), which remains a major cause of morbidity and mortality in this patient group [1, 2]. Dyslipidemia is an established independent risk factor for CVD. It is well known that in patients with end-stage renal disease (ESRD), changes in lipid metabolism occur, creating a complex form of dyslipidemia [3], and the lipid abnormalities persist or are aggravated during renal replacement treatment [4, 5], which may partly explain the high incidence of CVD [6].

In the past few years, there has been a growing scientific and public interest in the role of omega-3 fatty acids, mainly obtained from fish and fish oil in CVD, idiopathic IgA nephropathy, lupus nephritis and renal failure. By modulating cell membrane structure and function as well as synthesis of lipid mediators such as eicosanoids, omega-3 fatty acid supplementation may offer multiple health benefits to dialysis patients [7].

Numerous clinical trials concerning the effects of fish oil supplements on serum lipids in dialysis patients have been published. However, the results are still inconclusive, because the trials included small numbers of patients and different doses of fish oil, with short duration of observation. Therefore, we present the results of a systematic review summarizing current evidence from randomized controlled trials of the effects of fish oil supplements on serum lipid profile in dialysis patients.

## Methods

### Search strategy

The systematic review was conducted in accordance with PRSIMA guidelines [8]. The search used key words related to dialysis (kidney failure, chronic renal failure, dialysis, hemodialysis, peritoneal dialysis), omega-3 fatty acids (fatty acid, omega-3, fish oil, α-linolenic acid, eicosapentaenoic acid, docosahexanoic acid) and lipid (lipid, cholesterol, triglyceride, lipoprotein, hyperlipidemia) to identify randomized controlled trials published in the English language covering the period from as early as possible to October 2013, from different computerized databases including PubMed, EMBASE and the Cochrane Central Register of Controlled Trials. In addition, the reference lists of the published papers on clinical trials, review articles and meta-analysis were hand-searched for other relevant studies. The investigators were contacted for unreported lipid data in published trials. The titles and abstracts of the articles were analyzed to ascertain conformity with the inclusion criteria. The full text of an article was reviewed carefully if the screening of its title and abstract was unclear as to its admissibility. The complete search strategy is available in the Additional file 1.

### Selection criteria

We included published completed studies that enrolled dialysis patients, regardless of the type of dialysis. Randomized controlled trials were included if they met the following criteria: (i) the study compared fish oil (any dose or type) versus placebo/no treatment; (ii) concomitant therapy with fish oil equally in both treatment arms; and (iii) at least one of the following four outcomes were reported: serum TG, TC, LDL-C, HDL-C. All the studies were reviewed by two independent reviewers (Han Du, He Zhang) and any disagreement was resolved by discussion.

### Quality assessment

To comply with the Cochrane Handbook for Systematic Reviews of Interventions in terms of quality assessment of the randomized controlled trials, we evaluated the quality of the studies in terms of allocation concealment and intention-to-treat analysis, blinding of investigators, participants and outcome assessors, and completeness to follow-up, as well as the Jadad scale [9–11]. The Jadad scale was performed by two reviewers independently (Wei Zhu, Jie Chen).

### Data extraction

We followed a standard Cochrane protocol for study selection and data extraction [12, 13]. Study eligibility was determined by consensus, based on previously determined inclusion criteria. Eligible studies were reviewed independently by two authors (Wei Zhu, Feng Hu) who used data extraction forms developed for this purpose. We used consensus and a third reviewer (Chongya Dong), if necessary, to resolve disagreements. Data were extracted from all included trials in terms of baseline characteristics of the trials, type of dialysis, the components and dose of fish oil, follow-up duration, and the following reported outcomes: serum TG, TC, LDL-C, and HDL-C. If an outcome was reported at more than one time point in a single study, the longest period of follow-up was used.

### Data synthesis and statistical analysis

The mean difference in TG, TC, HDL-C and LDL-C from baseline was extracted into meta-analysis as a continuous variable. The effects of fish oil compared with control oil on the lipid profile were defined as the mean difference between the changes in serum lipid concentration. The mean changes were calculated by subtracting the baseline values from the final values. For studies only reporting data of the pre-intervention mean and SD and the post-intervention mean and SD, we inputted the missing SD of changes from baseline using a correlation coefficient of 0.7, as calculated and averaged based on studies with complete outcome reports [14–17]. For cross-over studies, the correlation coefficient was 0.797, as calculated based on a complete outcome report with cross-over design [18].

Heterogeneity among studies was assessed using Cochran’s *Q* test and *I*^*2*^ statistic. *P* <0.05 or *I*^2^ > 50% was considered significant. In that case, the random effects model was used as the pooling method. Otherwise, the fixed effect model was chosen. Funnel plots and Egger’s regression were used to assess the potential publication bias.

We excluded the study by Taziki after the data synthesis phase [19], and the correlation coefficient was not available in primary reports, therefore we performed a sensitivity analysis.

The components and dose of fish oil, duration of intervention, and initial lipid levels may influence the results, therefore we performed subgroup comparisons based on the parameters above. Because these previous studies measured fish oil as a whole and it has been reported that DHA and EPA content varied in different types of fish oil, we also subgrouped the trials on the basis of the dose of DHA + EPA in the included trials. Meta-regression was performed to compare the effects of these subgroup characteristics on the outcomes.

Review Manager 5.2 created by the Cochrane Collaboration for meta-analysis (http://www.cochrane.org) was used for statistical analysis.

### Role of the funding source

This study had no funding source. The corresponding author had full access to all the data in the study and had final responsibility for the decision to submit for publication.

## Results

### Literature search

**Figure 1:**
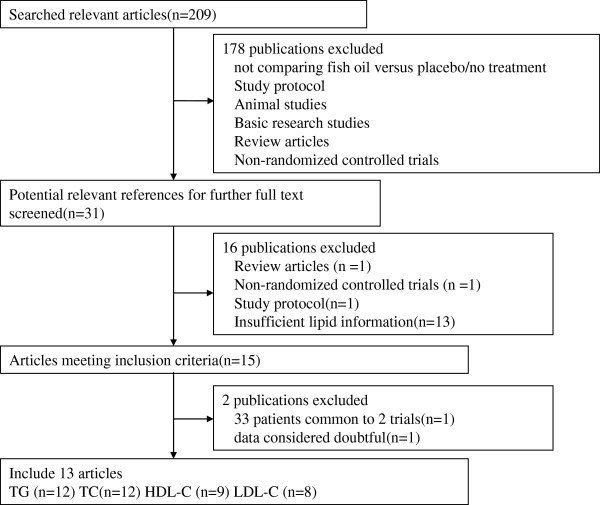
**Flow diagram of study selection process.**

A total of 209 articles were found in our initial search, and 178 were excluded by screening the titles or abstracts, because they were animal studies, basic research studies, review articles, or non-randomized controlled trials, and they did not enroll dialysis patients or compare fish oil versus placebo/no treatment. Full-text assessment of the 31 potentially relevant articles resulted in 13 eligible randomized controlled studies. The reasons for exclusion were as follows: one article was a review one trial was not randomized and controlled trial, one full-text was a study protocol, and 13 studies did not report sufficient lipid detail for inclusion in the meta-analysis. Two studies had the same first author, dialysis clinics, start and end point, inclusion and exclusion criteria [20, 21], and we confirmed that 33 patients had been included in both studies. In the absence of prompt availability of patient-level data for both trials, we only included the trial with the highest number of patients [20]. One trial was excluded because we found the data quality questionable. Unfortunately, we were unable to contact the author to confirm the data quality. Finally, all the authors agreed to exclude it after discussion. The reasons were as follows: (i) Fasting serum TC should be >220 mg/dl as per the inclusion criteria. However, the baseline levels provided for both groups (102 ± 32 and 229 ± 31 mg/dl) were not within the inclusion criteria [22]; (ii) The baseline TG level in the drug group was significantly higher than in the control group (321 ± 29 vs 268 ± 22 mg/dl), and the baseline TC level was significantly lower (102 ± 32 vs 229 ± 31 mg/dl). Both groups should have matching data. This could be the cause of the apparent benefit with fatty acid supplementation. The data regarding baseline TG of the control group in the two tables are different (268 ± 22 and 268 ± 32 mg/dl) [22]. We included the study by Poullia in spite of patients received different α-tocopherol doses as concomitant therapy [18], because the supplement aimed to control the possible effects of α-tocopherol present in Omacor capsules, and had no impact on lipid profile [23]. A flow diagram of the article selection for this meta-analysis is shown in Figure 1.

### Quality assessment

Quality assessment of the primary studies is summarized in Table 1. The Jadad scores ranged from 0 to 5 points. Study quality on the whole was good; five of 13 studies had a Jadad score of 5, four of 13 had a Jadad score of 3 or 4, and four of 13 had a Jadad score of ≤2. Participants and investigators were blinded in nine of 13 trials; sufficient details of drop-outs and withdrawals were described in 13 trials, two studies complied with allocation concealment; and 4 studies met the intention-to-treat analysis criteria.

**Table 1:** **Quality assessment of randomized, controlled trials included in the review**

Reference	Jadad score					Sum of Jadad score	Lost at follow-up	Intention to treat analysis	Allocation concealment
	Randomization	Appropriateness of randomization	Double blind	Appropriateness of double blind	Analysis reasons for withdrawals				
Diskin, 1990 [24]	1	0	0	0	1	2	3/7	no	unknown
Donnelly, 1992 [25]	1	0	1	1	1	4	3/16	yes	unknown
Ando, 1999 [26]	1	0	0	0	1	2	0/38	no	unknown
Khajehdehi, 2000 [27]	1	0	0	0	1	2	0/60	no	unknown
Schmitz, 2002 [28]	1	1	1	1	1	5	1/24	yes	unknown
Saifullah, 2007 [14]	1	1	1	1	1	5	4/27	no	unknown
Svensson 2008 [29]	1	1	1	1	1	5	54/206	no	adequate
Bowden, 2009 [20]	1	1	1	1	1	5	9/96	yes	unknown
Poulia, 2011 [18]	1	0	0	0	1	2	8/30	no	unknown
Kooshki, 2011 [15]	1	1	1	0	0	3	0/34	no	unknown
W.S. An, 2011 [30]	1	1	1	0	1	4	4/18	no	unknown
Lok, 2012 [16]	1	1	1	1	1	5	5/201	yes	adequate
Daud, 2012 [17]	1	0	1	1	1	4	9/63	yes	unknown

### Study characteristics

We identified 13 trials with 764 subjects in our study [14–18, 20, 24–30]. The characteristics of the trials are shown in Table 2. One study focused on hyperlipidemia patients. One study enrolled subjects with serum albumin ≤3.9 g/dl. Eleven of the trials were parallel trials and two were cross-over studies. Ten of the trials enrolled patients on hemodialysis, one trial on peritoneal dialysis, and two on both peritoneal dialysis and hemodialysis. Duration of fish oil supplementation was 4 weeks to 12 months. The daily amount of EPA + DHA supplement ranged from 0.9 g (dose of EPA + DHA was estimated as 60% of fish oil dose) to 3 g [16, 19]. All 13 studies reported the effects on TG levels; 12 studies reported the effects on cholesterol, and nine and eight studies investigated the effects of fish oil on HDL-C and LDL-C, respectively.

**Table 2:** **Characteristics of randomized, controlled trials included in the meta-analysis**

Reference	Additional information	Population	Sex(M + F)	Age	HD + PD	Duration	Treatment group	Control group
		F/C	F/C	F/C(SD or SE)	F/C			
Diskin, 1990 [24]	Unspecified	4/3	NA	NA	4 + 0/3 + 0	24 w	EPA (3 g/d)	placebo
Donnelly, 1992 [25]	1DM	14/15	5 + 3/7 + 1	52 (14)/51 (12)	4 + 4/4 + 4	4 w	fish oil (3.6 g/d) (12%DHA/18%EPA)	olive oil
Ando, 1999 [26]	HD:16DM/22, PD:0DM/16	19/19	17 + 2/16 + 3	54 (11)/51 (13)	11 + 8/11 + 8	3 m	EPA (1.8 g/d)	placebo
Khajehdehi, 2000 [27]	DM 15%	15/15	8 + 7/7 + 8	32.7 (10.7)/31.1 (8.8)	15 + 0/15 + 0	2 m	fish oil (1.5 g/d)	corn oil (4.5 g/d), seasame oil (4.5 g/d), placebo
Schmitz, 2002 [28]	Unspecified	12/12	5 + 7/6 + 6	52 (6)/54 (3)	12 + 0/12 + 0	3 m	fish oil (4 g/d)(24%DHA/44%EPA)	corn oil(4 g/d)
Saifullah, 2007 [14]	Unspecified	15/8	11 + 4/7 + 1	58 (12)/57 (14)	15 + 0/8 + 0	12 w	fish oil (DHA 170 mg/d × 2, EPA 340 mg/d × 2)	placebo (soybean/corn oil mixture)
Svensson 2008 [29]	Unspecified	35/38	NA	NA	35 + 0/38 + 0	3 m	omega-3 PUFA (1.7 g/d) (45%EPA/37.5%DHA)	olive oil
Bowden, 2009 [20]	Unspecified	44/43	25 + 19/20 + 23	59.3 (12.7)/60.8 (10.3)	44 + 0/43 + 0	6 m	fish oil (6 g/d) (EPA 0.96 g/d, DHA 0.6 g/d)	corn oil (6 g/d)
Poulia, 2011 [18]	Unspecified	22/23	NA	NA	22 + 0/23 + 0	4 w	omega-3 (EPA 920 mg/d, DHA 760 mg/d) + VitE 8 mg/d	VitE (14.2 mg/d)
Kooshki, 2011 [15]	Unspecified	17/17	10 + 7/11 + 6	50 (18)/50 (17)	17 + 0/17 + 0	10 w	omega-3 (2080 mg/d) (EPA 1240 mg/d, DHA 840 mg/d)	placebo (medium chain triglycerides oil)
W.S. An, 2011 [30]	Unspecified	7/7	4 + 3/3 + 4	51.6 (8.7)/52.6 (12.1)	0 + 7/0 + 7	12 w	fish oil (3 g/d)EPA 460 mg/d × 3 + 380 mg DHA/d × 3	corn oil
Lok, 2012 [16]	Unspecified	99/97	NA	NA	99 + 0/97 + 0	6 m,12 m	fish oil (4 g/d) (EPA 1600 mg/d, DHA 800 mg/d)	olive oil (3 g/d)
Daud, 2012 [17]	ALB < 3.9 g/dl	31/32	20 + 11/20 + 12	59 (13)/58 (12)	31 + 0/32 + 0	24 w	protein + omega-3 (2.4 g) (EPA 1800 mg, DHA 600 mg), three times a week	protein + olive oil, three times a week

DM, diabetes; HD, hemodialysis; PD, peritoneal dialysis; NA, not available; F, fish oil group; C, control group.

### Effects of fish oil supplementation on lipid concentrations

**Figure 2:**
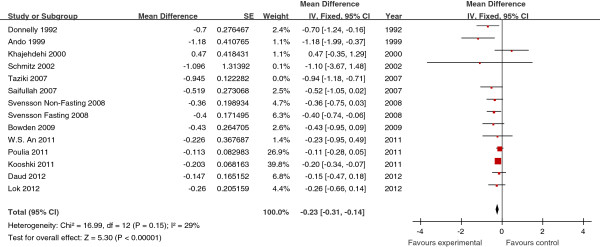
**Forest plots depicting the effect of fish oil supplement on TG.** IV, inverse variance; fixed, fixed effects model; CI, confidence interval.

**Figure 3:**
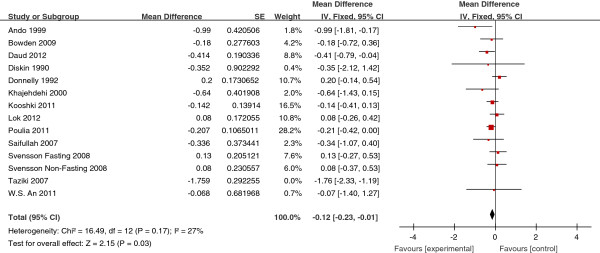
**Forest plots depicting the effect of fish oil supplement on TC.** IV, inverse variance; fixed, fixed effects model; CI, confidence interval.

**Figure 4:**
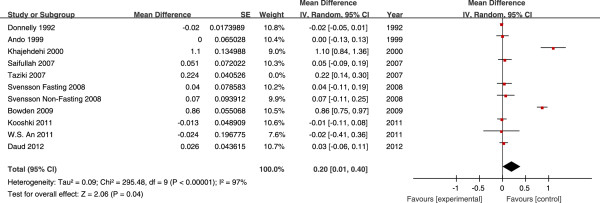
**Forest plots depicting the effect of fish oil supplement on HDL-C.** IV, inverse variance; fixed, fixed effects model; CI, confidence interval.

**Figure 5:**
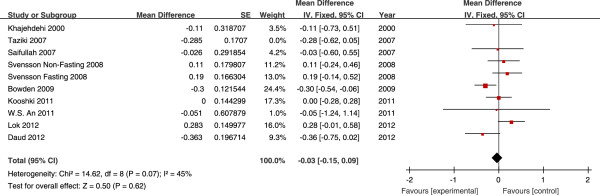
**Forest plots depicting the effect of fish oil supplement on LDL-C.** IV, inverse variance; fixed, fixed effects model; CI, confidence interval.

The effects of fish oil on the levels of TG, TC, HDL-C and LDL-C in all studies are shown in Figures 2, 3, 4 and 5, respectively. Overall, the pooled analysis of the effects of fish oil intake on TG levels showed a decrease of 0.23 mmol/L compared with the control group (95% CI, −0.31, −0.14, *P* <0.01) (Figure 2). Fish oil also significantly lowered serum TC levels by 0.12 mmol/L (95% CI, −0.23, −0.01, *P* =0.03) (Figure 3). Fish oil significantly increased HDL-C levels by 0.20 mmol/L (95% CI, 0.01, 0.40, *P* <0.01). Heterogeneity was observed for the HDL-C outcome (heterogeneity chi-square = 836.86, *I*^*2*^ = 97%, *P* = 0.04) (Figure 4). Fish oil did not have any significant influence on LDL-C (mean difference −0.03 mmol/L; 95% CI, −0.15, 0.09, *P* = 0.62) (Figure 5).

### Publication bias

**Figure 6:**
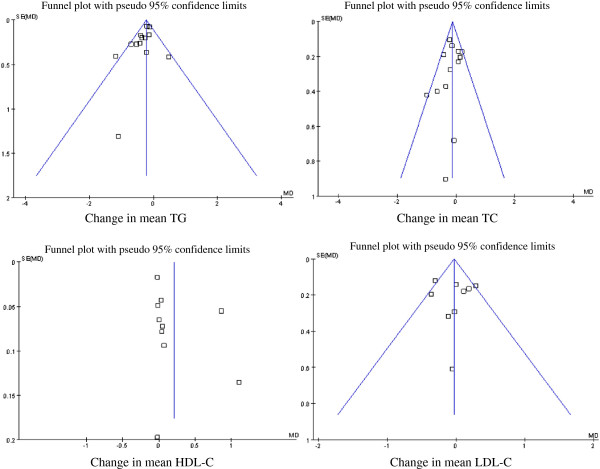
**Funnel plots of studies included in meta-analysis on the effects of fish oil on serum lipid parameters.** The results show potential publication bias for HDL-C, but not for other parameters.

The potential publication bias was detected by funnel plots and Egger’s regression test (Figure 6). The results suggested no publication bias for the effects of fish oil on the parameters, including TC, TG and LDL-C. However, funnel plots revealed that publication bias existed for HDL-C, which was also illustrated by Egger’s regression test (*P* <0.01). It may have been caused by two articles whose results deviated from the others [20, 27], and negative results about HDL-C are published less often.

### Subgroup analyses

The results of the subgroup analyses are shown in Table 3. The effects of fish oil on serum TG were found to be greater in patients with higher baseline TG levels. The mean change in TG in the subgroups with baseline levels ≥2.26, 1.69-2.26 and ≤1.69 mmol/L was −0.56, −0.18 and −0.24 mmol/L, respectively. However, no significance could be found in meta-regression analysis (*P* =0.75).

**Table 3:** **Result of subgroup analyses**

Subgroup		TG			TC			HDL-C			LDL-C		
		Effect size	95% CI	***I*** ^***2***^(%)	Effect size	95% CI	***I*** ^***2***^(%)	Effect size	95% CI	***I*** ^***2***^(%)	Effect size	95% CI	***I*** ^***2***^(%)
Baseline TG (mmol/L)	≥2.26	**−0.56**	**−0.95, −0.17**	66	−0.06	−0.36, 0.23	78	0.33	−0.07, 0.74	97	**−0.11**	−**0.73, 0.51**	-
	1.69-2.26	**−0.18**	**−0.30, −0.05**	0	**−0.15**	**−0.30, −0.01**	20	0.32	−0.27, 0.91	99	−0.08	−0.23, 0.07	76
	≤1.69	**−0.24**	**−0.36, −0.12**	0	−0.08	−0.29, 0.13	0	0.01	−0.06, 0.08	0	7	−0.13, 0.26	0
Baseline TC (mmol/L)	>5.17	**−0.70**	**−1.24, −0.16**	-	0.18	−0.15, 0.51	0	−0.02	−0.05, 0.01	-	−0.03	−0.15, 0.09	45
	≤5.17	**−0.22**	**−0.30, −0.13**	21	**−0.16**	**−0.28, −0.04**	21	0.23	−0.02, 0.48	97	-	-	-
Component of fish oil	EPA	**−1.18**	**−1.99, −0.37**	-	−0.88	−1.62, 0.13	0	0.00	−0.13, 0.13	-	-	-	-
	EPA + DHA	**−0.22**	**−0.31, −0.14**	0	−0.09	−0.21, 0.02	12	**0.23**	**0.01, 0.44**	97	-	-	-
Dose of EPA + DHA	<2 g	**−0.24**	**−0.35, −0.13**	45	−0.15	−0.28, −0.02	40	**0.23**	**0.01, 0.45**	97	−0.11	−0.26, 0.03	33
	≥2 g	**−0.21**	**−0.34, −0.08**	0	−0.06	−0.27, 0.15	0	**−0.20**	**0.01, 0.40**	-	0.14	−0.07, 0.34	46
Duration	≤12w	**−0.23**	**−0.32, −0.14**	44	−0.11	−0.24, 0.02	37	0.12	−0.01, 0.26	90	0.06	−0.10, 0.23	0
	>12w	**−0.24**	**−0.46, −0.01**	0	−0.15	−0.38, 0.07	20	0.44	−0.37, 1.26	97	−0.12	−0.29, 0.04	82

The bold number is to emphasize the effects (and 95% CI) that are significant.

Long-term (>12 w) intervention demonstrated a tendency towards greater improvement in serum HDL-C and LDL-C levels compared with short-term intervention (≤12 w). The mean change in HDL-C in the ≤12 w and >12 w subgroups was 0.12 and 0.44 mmol/L, respectively, and the change in LDL-C in ≤12 w and >12 w subgroups was 0.06 and −0.12 mmol/L, respectively. However, meta-regression analysis showed no significant association between serum HDL-C or LDL-C outcomes and duration of intervention (*P* =0.12, *P* =0.31). There was no significant difference in the effects between doses and components of fish oil on lipid levels.

### Sensitivity analysis

For sensitivity analysis, because we used a correlation coefficient of 0.7 to input the missing SD of changes from baseline as calculated and averaged based on studies with complete outcome reports, we also inputted correlation coefficients of 0.5 and 0.9 [31]. This had no effect on the significance of the pooled changes in TG, TC, LDL-C and HDL-C.

Although the trial by Taziki was excluded [19], we also analyzed the results that included Taziki’s trial data. We compared the results when we included and excluded Taziki’s trial data. The significance of the pooled changes in TG, TC, LDL-C and HDL-C was not altered, except that including the Taziki data changed the homogeneity of the TG and TC studies (*I*^*2*^: TG 29 to 73%, TC 27 to 72%).

The results of two studies deviated from others obviously, which may be the reason for the positive publication bias result for HDL-C [20, 27]. Therefore, we analyzed the results after excluding these data, but reached the same conclusion as before. Fish oil did not have any significant influence on HDL-C (mean difference −0.01 mmol/L; 95% CI, −0.03, 0.02 mmol/L, *P* =0.90).

## Discussion

Our meta-analysis showed supplementation with fish oil was associated with a decrease in TG and TC, and an increase in HDL-C, but had no significant effects on LDL-C compared with controls. Funnel plots and Egger’s regression test showed possible publication bias for HDL-C, but not for other parameters.

The results of the current meta-analysis on TC, TG, LDL-C and HDL-C were similar to those of Pei [32], in which fish oil was found to reduce serum TG levels significantly by −0.78 mmol/L (95% CI: −1.12, −0.44 mmol/L, *P* <0.01), but had no significant effect on TC, HDL-C and LDL-C compared with controls. However, there were several differences between the two studies. The two studies by Svensson and Bouzidi [33, 34] were included in the study of Pei, but were excluded in the current study, because the patients enrolled in the two studies did not undergo dialysis treatment. We were able to confirm that two studies of Bowden were based on the same population [20, 21], so we excluded the smaller sample study [21]. The trial by Taziki was excluded in our meta-analysis [19], but included in the analysis of Pei. We also added seven studies not included in Pei’s review because of small sample sizes (<15 patients in either arm) and search dates [15–18, 24, 29, 30]. Furthermore, we added several subgroup analyses based on components of fish oil and initial lipid levels.

Subgroup analyses revealed good long-term trends in the effects of supplements of fish oil on serum HDL-C and LDL-C levels. However, as mentioned before, the degree of improvement did not achieve statistical significance, which may have been due to a lack of statistical power. If we want to clarify the long-term effect, more specific studies should be conducted.

There are two issues that need to be carefully scrutinized. The first is that the optimal daily dose in dialysis patients that has a lipid-altering effect is still not established. In our study, the content of fish oil supplements varied significantly. It is impossible to determine the precise content of each supplement, thus the dose of DHA and EPA varied, so we used the dose of EPA + DHA for subgroup analysis. We found no difference in the effects on lipid profile when comparing <2 g EPA + DHA supplement with ≥2 g, therefore a high dose of fish oil supplement for lowering lipid levels might not be needed. As a result of dietary restrictions on fish consumption, to control phosphorus intake, dialysis patients have significantly lower levels of omega-3 fatty acids in their erythrocyte membranes [35], and based on randomized controlled trials, we suggest that the daily supplement dose of fish oil for dialysis patients should be >1 g. This is different from the current recommended dose of up to 1 g/day, by the American Heart Association (AHA) and various international health organizations [36, 37].

Second, the truly active component of fish oil accounting for the lipid-altering effects is still unclear. After a period of DHA supplementation, Harrison reported no significant changes in LDL-C or TC, and a concomitant increase in HDL-C. The authors further speculated that only EPA has lipid-modulating effects [38]. We tried to establish whether EPA and DHA had a different impact on lipid profile through subgroup analyses. Our study confirmed that EPA had a significant influence on lipid profile, and EPA + DHA showed the same lipid-modulating effects as EPA alone. Unfortunately, we could not draw any conclusion about the effect of DHA alone on lipid profile in dialysis patients.

The lipid-altering effects of fish oil may be attributed to many factors [7]. Cell membrane fatty acids play an important role in signal transduction, therefore omega-3 fatty acids are capable of modifying gene expression. It is believed that the dramatic lipid-altering effects of omega-3 fatty acids are mediated via this mechanism [39]. Specifically, omega-3 fatty acids modulate the function of peroxisome proliferator-activated receptors and sterol regulatory binding proteins, both of which are involved in lipid homeostasis [40, 41].

Although fish oil is generally well tolerated, attention should be paid to the safety of fish oil supplementation at doses >1 g/day. Most adverse effects involve a transient minimal decrease in the desire for food [27], gastrointestinal complaints (e.g. gas, bloating, fishy aftertaste [28], nausea and burping [14]), and prolonged bleeding time [25]. Serious bleeding complications were restricted to a single patient in one study [24]. However, the majority of studies were not designed to measure side effects and tolerability after long-term use.

CVDs are the most important cause of mortality in patients undergoing hemodialysis. The frequency of CVD in dialysis patients has been reported as 3–45 times that observed in the general population and 50% of deaths in these patients are related to CVD [42]. Lipid abnormalities often complicate chronic renal failure and persist or are aggravated during renal replacement treatment [4, 43]. Large cohort studies have shown an inverse association between cardiovascular morbidity and mortality and fish oil ingestion [44, 36]. Lok found that among patients with new hemodialysis grafts, daily ingestion of fish oil improved cardiovascular event-free survival [16]. Friedman showed that long-chain omega-3 fatty acids are strongly and independently associated with a lower risk of sudden cardiac death in hemodialysis patients throughout the first year of dialysis [45]. Except for lipid modulation, fish oil may reduce cardiovascular events by several mechanisms, including anti-inflammatory, antiarrhythmic and plaque-stabilizing effects as well as improved endothelial effects [46, 47]. However the potential benefits of fish oil on cardiovascular events deserve confirmation in future studies.

There were some strengths and limitations to our study. We had more strict inclusion criteria, and analyzed almost all the lipid parameters mentioned in the literature. A series of important subgroup analyses were also performed. However, the following limitations must be mentioned. First, none of the trials was sufficiently powered because of the small numbers of participants. Second, until now, there has been insufficient evidence for the effectiveness of long-term supplementation with fish oil on lipid profile, because the longest duration in these trials was only 48 weeks [12]. Third, publication bias is a major potential limitation of meta-analysis that has been minimized by contacting investigators for unpublished results. Funnel plots and Egger’s regression test showed possible publication bias for HDL-C, and sensitivity analysis found that the significance of the change in HDL-C level was altered. We must interpret the HDL result with caution. Finally, none of the studies were specifically designed to assess the effects of fish oil in dyslipidemia patients, thus it was not accurate to evaluate the lipid-altering effects in this group. Based on the above, the results derived from this meta-analysis should be treated with considerable caution.

## Conclusions

This meta-analysis suggests that fish oil reduces serum TG and TC levels, and increases HDL-C levels, without influencing LDL-C levels among patients undergoing dialysis treatment. This effect may directly account for part of the observed benefits of fish intake on cardiovascular risk. Based on randomized controlled trials, we suggest that the daily supplement dose of fish oil for dialysis patients should be >1 g, but a high dose might not be necessary. Future large-scale randomized trials with adequate doses and duration of treatment are needed to determine the effect of fish oil supplements on lipid profile in dialysis patients, especially those with CVD.

## Electronic supplementary material

Additional file 1: **PubMed search strategy.** (PDF 6 KB)
