# Supplementary material for: Effects of fish oil on serum lipid profile in dialysis patients: a systematic review and meta-analysis of randomized controlled trials
Source: Lipids Health Dis. 2014 Aug 8;13:127. doi: 10.1186/1476-511X-13-127 (PMC4266905; doi:10.1186/1476-511X-13-127)
Supplement: Supplementary file 1 — Additional file 1: PubMed search strategy. (PDF 6 KB) [file 12944_2014_1118_MOESM1_ESM.pdf]

Additional file 1 : PubMed search strategy

Database: PubMed from as early as possible to October 2013

Search Strategy:

("omega-3 fatty acids"[All Fields] OR "fatty acid"[All Fields] OR "omega-3"[All Fields] OR "fish oil"[All Fields] OR "a-linolenic acid"[All Fields] OR "eicosapentanoic acid"[All Fields] OR "docosahexanoic acid"[All Fields]) AND ("kidney failure"[All Fields] OR "chronic renal failure"[All Fields] OR ("renal dialysis"[MeSH Terms] OR ("renal"[All Fields] AND "dialysis"[All Fields]) OR "renal dialysis"[All Fields] OR "dialysis"[All Fields] OR "dialysis"[MeSH Terms]) OR ("haemodialysis"[All Fields] OR "renal dialysis"[MeSH Terms] OR ("renal"[All Fields] AND "dialysis"[All Fields]) OR "renal dialysis"[All Fields] OR "hemodialysis"[All Fields]) OR "peritoneal dialysis"[All Fields]) AND (("lipids"[MeSH Terms] OR "lipids"[All Fields] OR "lipid"[All Fields]) OR ("cholesterol"[MeSH Terms] OR "cholesterol"[All Fields]) OR ("triglycerides"[MeSH Terms] OR "triglycerides"[All Fields] OR "triglyceride"[All Fields]) OR ("lipoproteins"[MeSH Terms] OR "lipoproteins"[All Fields] OR "lipoprotein"[All Fields]) OR ("hyperlipidaemia"[All Fields] OR "hyperlipidemias"[MeSH Terms] OR "hyperlipidemias"[All Fields] OR "hyperlipidemia"[All Fields])) AND (Clinical Trial[ptyp] AND "humans"[MeSH Terms]) AND English[LA]
